# Innovative Design of Bismuth-Telluride-Based Thermoelectric Transistors

**DOI:** 10.3390/ma16165536

**Published:** 2023-08-09

**Authors:** Hao Deng, Bohang Nan, Guiying Xu

**Affiliations:** School of Materials Science and Engineering, University of Science and Technology Beijing, Beijing 100083, China

**Keywords:** Bi_2_Te_3_, thermoelectric transistor, output performance, theoretical calculation

## Abstract

Conventional thermoelectric generators, predominantly based on the π-type structure, are severely limited in their applications due to the relatively low conversion efficiency. In response to the challenge, in this work, a Bi_2_Te_3_-based thermoelectric transistor driven by laser illumination is demonstrated. Under laser illumination, a temperature difference of 46.7 °C is produced between the two ends of the transistor structure. Further, the hole concentrations in each region redistribute and the built-in voltages decrease due to the temperature difference, leading to the formation of the transistor circuit. Additionally, the operation condition of the thermoelectric transistor is presented. The calculation results demonstrate that the maximum output power of such a designed thermoelectric transistor is 0.7093 μW.

## 1. Introduction

Fossil fuels, being the primary and nonrenewable energy source that has driven human society and industrial development since the Industrial Revolution, are facing an inevitable depletion due to the ever-growing demand. The resulting concerns over energy security have also contributed to escalated global conflicts [1]. Therefore, it is imperative to focus on the development of clean, low-carbon, secure, and efficient renewable energy sources.

Thermoelectric generators, capable of converting heat into electricity through the Seebeck effect, have emerged as a promising energy conversion technology [2]. Conventional thermoelectric generators typically consist of multiple thermoelectric modules, offering numerous advantages, such as high safety, extended service life, zero waste generation, no noise, and simple structure [3]. However, the applications of conventional thermoelectric generators are limited due to their relatively poor output performance. Therefore, thermoelectric generators can only be used in a few specific scenarios, such as medicine, aerospace, and military sectors.

In general, the output performance of thermoelectric generators was assessed based on the *zT* value of materials, output power, and conversion efficiency. To enhance the output performance, the combination of thermoelectric generators with transistor technology has been proposed [4,5]. Bejenari et al. studied the thermoelectric performance of Bi_2_Te_3_ nanowires in the transistor structure under the gate voltage. Theoretical results indicated that the *zT* value could reach 3.4 for Bi_2_Te_3_ nanowires [6]. Subsequently, Qin et al. found that the *zT* values of N-type and P-type Bi_2_Te_3_ films could reach 1.22 and 1.02 in the transistor structure through experimental measurement [7]. Furthermore, Nan et al. studied the output performance of a thermoelectric transistor driven by the Seebeck effect, in which the built-in electric field is perpendicular to the gradient temperature field. Results showed that the output power was 17.8 mW and conversion efficiency could reach 8.69% at a temperature difference of 50 °C [8,9].

These research results demonstrated that the output performance of thermoelectric generators could be significantly improved through the transistor effect. In this work, a Bi_2_Te_3_-based thermoelectric transistor driven by the laser illumination is presented. On the one hand, the short pulse width of a laser allows for rapid creation of a temperature difference between the two ends of the device, reaching nanosecond levels [10]. On the other hand, Bi_2_Te_3_-based materials exhibit excellent thermoelectric properties at room temperature [11]. Moreover, utilizing Bi_2_Te_3_-based materials as research subjects can improve the accuracy of theoretical calculations due to their comprehensive performance parameters [12,13]. As a result, in this work, theoretical results indicated that the maximum output power of a single Bi_2_Te_3_-based thermoelectric transistor could reach 0.7093 μW with a temperature difference of 46.7 °C. Conventional thermoelectric generators would require multiple thermoelectric modules connected in series to achieve such output performance [14,15,16].

## 2. Theoretical Foundations

**Figure 1:**
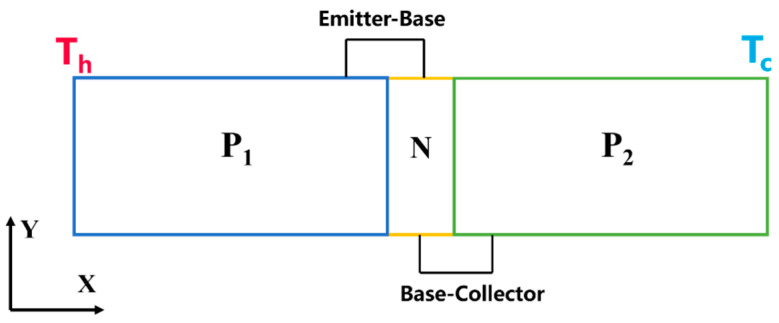
Diagram of thermoelectric transistor in X–Y plane.

The structure of the designed structure is depicted in the following Figure 1.

Considering the one-dimensional model, the length (*x*_1_) of P_1_ region (P_1_-Bi_2_Te_3_) is 1 μm, while the length of (*x*_2_) P_2_ region (P_2_-Si) is 4 μm. The scale of N region (N-Si) is the nanometer range, which can be neglected for calculation purposes.

Using a laser as the heat source, serving as the triggering condition, the P_1_-Bi_2_Te_3_ is irradiated by the laser at one end, causing its temperature to rise. Subsequently, the heat is transmitted throughout the entire structure via thermal conduction, forming a temperature field along the X direction. The temperature field induces the redistribution of majority charge carriers (holes) within the structure. The holes in P_1_ region and P_2_ region diffuse from the hot end to the cold end, accumulating at the cold end. Simultaneously, the built-in voltage across P_1_–N junction decreases from the cold end to the far end. Consequently, the balance between the diffusion potential and drift potential on both sides of P_1_–N junction is disrupted, leading to the injection of holes from P_1_ region into N-type region at the cold end. Additionally, since the length of N-type region is on the nanoscale, the holes further migrate towards P_2_ region. Subsequently, the accumulated holes in P_2_ region flow back to the N-type region along the wires. The migration of holes is consistent with the common base circuit of a bipolar transistor. Thus, it is inferred that the transistor circuit can be formed in the PNP structure under laser irradiation. In other words, the cold side of the P_1_ region (*x*_1_) functions as the emitter of the thermoelectric transistor, while the hot side of the P_2_ region (*x*_2_) serves as the collector, with the N-type region acting as the base [17].

### 2.1. Temperature Distribution of Bi_2_Te_3_ and p-Type Si under Laser Irradiation

#### 2.1.1. Calculation of Temperature Distribution in P_1_-Bi_2_Te_3_

When a laser is incident upon Bi_2_Te_3_, according to Lambert’s law, it is known that the amount of light absorbed is closely related to the position in the one-dimensional model. The closer the position is to the source of light, the greater the light absorption. Assuming material thermal capacitance is uniform, a temperature gradient will form parallel to the direction of illumination. According to Fourier’s law, heat conduction will also occur along the direction of the incident light. Taking all these considerations into account under the one-dimensional condition, the following equation can be derived [18]:

(1) $$\rho c\frac{\partial T_{1}}{\partial t}=\frac{\partial }{\partial x}(k\frac{\partial T_{1}}{\partial x})+\left(1-R\right)\alpha I_{0}e^{-\alpha x}$$

where *ρ* represents material density, *c* represents material-specific heat capacity, *T*_1_ is the temperature, *k* represents thermal conductivity, *R* represents reflectivity, *I*_0_ represents light intensity, *t* represents time, *x* is the material length along the direction of illumination, and *α* represents absorption coefficient. 

Due to the extremely short duration of the pulse laser, it can be assumed that there is no heat transfer occurring at the ends of the sample. Therefore, the following conditions hold:

(2) $$\frac{\partial T_{1}\left(x=0,t\right)}{\partial x}=0$$

(3) $$\frac{\partial T_{1}\left(x=l,t\right)}{\partial x}=0$$

In addition, the initial temperature distribution of the sample is assumed to be uniform, with no heat transfer occurring between different parts of the sample. In other words, the temperature at each position is a constant, given by:

(4) $$T_{1}(x,0)=\varphi_{1}(x)=constant$$

By solving these equations in MATLAB R2020a, the temperature distribution *T*_1_(*x*_1_) in P_1_-Bi_2_Te_3_ can be obtained.

#### 2.1.2. Calculation of Temperature Distribution in P_2_-Si

Unlike the calculation of temperature distribution in Bi_2_Te_3_, the differential equation for the temperature distribution in P_2_-Si does not include the influence of illumination; thus, it becomes a one-dimensional heat conduction problem without a heat source:

(5) $$\rho c\frac{\partial T_{2}}{\partial t}=\frac{\partial }{\partial x}(k\frac{\partial T_{2}}{\partial x})$$

Similarly, the boundary conditions can be set as follows:

(6) $$\frac{\partial T_{2}\left(x=0,t\right)}{\partial x}=0$$

(7) $$\frac{\partial T_{2}\left(x=l,t\right)}{\partial x}=0$$

The initial condition for the P_2_-Si sample is:

(8) $$T_{2}(x,0)=\varphi_{2}(x)=constant$$

By solving these equations in MATLAB R2020a, the temperature distribution *T*_2_(*x*_2_) in P_2_-Si can be obtained.

### 2.2. Hole Concentration Distribution in Bi_2_Te_3_ and P_2_-Si

#### 2.2.1. Hole Concentration Distribution in P_1_-Bi_2_Te_3_

When P_1_-Bi_2_Te_3_ is subjected to a stable temperature field at 20 °C, the hole concentration is uniformly distributed, which is equal to the acceptor concentration *P_a_*. Subsequently, when P_1_-Bi_2_Te_3_ is influenced by a temperature gradient (*dx*/*dt* ≠ 0), the holes redistribute, while the acceptor ions remain fixed. The redistribution of holes can be obtained through theoretical analysis, as shown below.

DC transport equation for current [19]:

(9) $$J_{p}(x)=\sigma_{p}(x)\left[E\left(x\right)-S_{p}\left(x\right)\nabla T\left(x\right)\right]$$

(10) $$E\left(x\right)=\frac{d\varphi \left(x\right)}{dx}$$

where *J_p_*(*x*) represents the current density, ∇*T*(*x*) is the temperature gradient, *E*(*x*) denotes the electric field, *φ*(*x*) represents the electric potential, *σ_p_*(*x*) is the electrical conductivity of P_1_-Bi_2_Te_3_, and *S_p_*(*x*) is the Seebeck coefficient p-type Bi_2_Te_3_.

One-dimensional Poisson equation [20]:

(11) $$\frac{d^{2}\varphi \left(x\right)}{dx^{2}}=-\frac{e}{\varepsilon_{r}\varepsilon_{0}}\left[p_{1}\left(x_{1}\right)-P_{a}\right]$$

where *e* is the electronic charge, *ε_r_* is the relative permittivity, *ε*_0_ is the vacuum permittivity, *p*_1_(*x*_1_) represents the hole concentration distribution in P_1_-Bi_2_Te_3_, and *P_a_* is the constant concentration of acceptor holes assuming complete ionization of Bi_2_Te_3_.

Continuity equation [21]:

(12) $$\frac{\partial p_{1}\left(x_{1}\right)}{\partial t}=-\frac{1}{q}\frac{\partial J_{p}\left(x\right)}{\partial x}+\left(G_{p}-R_{p}\right)$$

where *G_p_* is the hole carrier generation rate in Bi_2_Te_3_, and *R_p_* is the hole carrier recombination rate.

Since P_1_-Bi_2_Te_3_ discussed in this work is a degenerate semiconductor, *σ_p_*(*x*) and *S_p_*(*x*) can be expressed as [11,22]:

(13) $$\sigma_{P}\left(x\right)=p\left(x\right)e\mu_{p}$$

(14) $$S_{p}\left(x\right)=\left[\frac{8\pi^{\frac{8}{3}}m_{p}^{*}{k_{B}}^{2}\left(r+\frac{3}{2}\right)}{3^{\frac{5}{3}}h^{2}e}\right]\frac{T_{1}\left(x_{1}\right)}{{p_{1}\left(x_{1}\right)}^{\frac{2}{3}}}$$

where *μ_p_* represents the hole mobility of P_1_-Bi_2_Te_3_, *r* is the scattering factor (= −1/2), *h* is the Planck constant, $m_{p}^{*}$ denotes the effective mass, and *T*_1_(*x*_1_) is temperature distribution of P_1_-Bi_2_Te_3_ along the *x* direction.

Since the simulated temperature distribution is significantly lower than the intrinsic excitation temperature of Bi_2_Te_3_, it can be assumed that *G_p_* = *R_p_* and the total hole concentration in Bi_2_Te_3_ remains constant after applying the temperature gradient:

(15) $$\int_{0}^{l_{x}}P_{a}dx=\int_{0}^{l_{x}}p_{1}\left(x_{1}\right)dx$$

Substituting the above equation into Equation (10) can obtain:

(16) $$E\left(x\right)=\frac{d\varphi \left(x\right)}{dx}=\int_{0}^{l_{x}}\frac{-e}{\varepsilon_{r}\varepsilon_{0}}\left[p_{1}\left(x_{1}\right)-P_{a}\right]dx=0$$

Substituting Equation (16) into Equation (9) yields:

(17) $$J_{p}\left(x\right)=-\sigma_{p}\left(x\right)S_{p}\left(x\right)\nabla T\left(x\right)$$

Furthermore, assuming that the entire P_1_-Bi_2_Te_3_ reaches a stable state after the temperature distribution is established, Equation (12) can be written as:

(18) $$\frac{\partial p}{\partial t}=-\frac{1}{q}\frac{\partial J_{p}\left(x\right)}{\partial x}=0$$

By solving these equations in MATLAB, the hole concentration distribution of P_1_-Bi_2_Te_3_ can be obtained under a temperature gradient.

#### 2.2.2. Hole Concentration Distribution in P_2_-Si

Since the carrier concentration of P_2_-Si studied in this work is less than 10^18^ cm^−3^, it is considered a nondegenerate semiconductor. Therefore, the Seebeck coefficient can be expressed as [23]:

(19) $$S_{p}\left(x\right)=\frac{k_{B}}{e}\left[\left(r+\frac{5}{2}\right)+\ln\frac{2{\left(2\pi m^{*}k_{B}T_{2}\left(x_{2}\right)\right)}^{3/2}}{h^{3}n}\right]$$

Then, based on the temperature distribution *T*_2_(*x*_2_) in P_2_-Si and Equations (13), (15), (17), and (18), the hole concentration distribution of P_2_-Si can be derived.

### 2.3. Operation Conditions of Thermoelectric Transistor

In order for the thermoelectric transistor to operate properly, it must be in a forward-active mode [17]. Since the formation for the circuit of the thermoelectric transistor is only based on the Seebeck effect, the potential within it can be represented by the concentration distribution in each region. Therefore, it is crucial to determine appropriate concentration ranges within each region to ensure the normal operation of the thermoelectric transistor.

Firstly, the thermoelectric transistor is composed of P_1_-Bi_2_Te_3_, N-Si, and P_2_-Si materials. Before applying a temperature gradient (the entire transistor is at *T* = 20 °C), the respective donor or acceptor doping ion concentrations must be greater than their intrinsic carrier concentrations. Mathematically, it can be expressed as follows:

(20) $$P_{a1}>p_{i1}\left(T=20\;{}^{\circ}C\right)$$

(21) $$N_{d}>n_{i}\left(T=20\;{}^{\circ}C\right)$$

(22) $$P_{a2}>p_{i2}\left(T=20\;{}^{\circ}C\right)$$

where *P_a_*_1_, *N_d_*, and *P_a_*_2_ represent the doping concentrations in P_1_ region (P_1_-Bi_2_Te_3_, emitter), N region (N-Si, base), and P_2_ region (P_2_-Si, collector), respectively. *p_i_*_1_, *n_i_*, and *p_i_*_2_ represent the intrinsic carrier concentrations in the respective regions at *T* = 20 °C.

#### 2.3.1. Implementation of Forward Bias and Forward Conduction at the Emitter–Base 

After applying a temperature gradient, the emitter (P_1_ region) and the base (N region) should simultaneously satisfy the forward bias and forward conduction. The temperature distribution *T*_1_(*x*_1_) between P_1_ region and N region can be obtained from the previous section. According to the relevant literature [24], the built-in voltage at the emitter–base junction varies with temperature:

(23) $$V_{bi1}\left(x_{1}\right)=\frac{k_{B}T_{1}\left(x_{1}\right)}{q}\left[\ln\frac{P_{a1}}{p_{i1}\left(x_{1}\right)}+\ln\frac{N_{d}}{n_{i}\left(x_{1}\right)}\right]$$

where *k_B_* is the Boltzmann constant, *p_i_*_1_(*x*_1_) is the intrinsic carrier concentration affected by the temperature distribution *T*_1_(*x*_1_), while *P_a_*_1_ and *N_d_* remain constant regardless of the applied temperature gradient. The variations in *p_i_*_1_(*x*_1_) and *n_i_*(*x*_1_) with the temperature distribution can be described by the following [25,26]:

(24) $$p_{i1}\left(x_{1}\right)={\left(\frac{m_{p1}^{*}}{m_{e}}\right)}^{\frac{3}{2}}\times ({\frac{T_{1}\left(x_{1}\right)}{300})}^{\frac{3}{2}}\times \exp\left(-\frac{E_{gp1}}{T_{1}\left(x_{1}\right)}\right)\times 2.5\times 10^{19}$$

(25) $$n_{i}\left(x_{1}\right)=5.23\times 10^{15}\times {T_{1}\left(x_{1}\right)}^{\frac{3}{2}}\times \exp\left(\frac{6395.39}{T_{1}\left(x_{1}\right)}\right)$$

where $m_{p1}^{*}$ is the effective mass of the emitter, *m_e_* is the electron mass, and *E_gp_*_1_ is the bandgap of the emitter.

To achieve forward bias and forward conduction at the emitter–base junction, the built-in voltage *V_bi_*_1_(*x*_1_) should satisfy:

(26) $$V_{bi1}\left(x_{1}\right)>0$$

Additionally, after applying the temperature gradient, the potential of the emitter should be higher than that of the base:

(27) $$\varphi_{p1}\left(x_{1}\right)>\varphi_{n}\left(x_{1}\right)$$

Since the potential of each region in this work is mainly determined by its carrier concentration, Equation (27) can be written as:

(28) $$p_{1}\left(x_{1}\right)>N_{d}$$

where *p*_1_(*x*_1_) is the hole concentration distribution of the emitter after applying the temperature gradient. However, the concentration of the base remains constant (*N_d_*) because it is completely depleted. 

Finally, in order to ensure the forward conduction in the thermoelectric transistor, the generated voltage due to the Seebeck effect (the Seebeck voltage *V_s_*_1_(*x*_1_)) should be greater than the built-in voltage *V_bi_*_1_*(x*_1_). In other words, the emitter voltage *V_e_*(*x*_1_) should be greater than 0:

(29) $$V_{e}\left(x_{1}\right)=V_{s1}\left(x_{1}\right)-V_{bi1}\left(x_{1}\right)>0$$

(30) $$V_{s1}\left(x_{1}\right)=∆T\left(x_{1}\right)\times S_{1}$$

(31) $$S_{1}=\left[\frac{8\pi^{\frac{8}{3}}m_{p1}^{*}k_{B}^{2}\left(r+\frac{3}{2}\right)}{3^{\frac{5}{3}}h^{2}e}\right]\left[\frac{T_{1}\left(x_{1}\right)}{{p_{1}\left(x_{1}\right)}^{\frac{2}{3}}}\right]$$

where *S*_1_ represents the Seebeck coefficient of the emitter and ∆*T*(*x*_1_) is the temperature distribution between the emitter and the base. By combining these equations, the forward bias and forward conduction at the emitter–base can be achieved.

#### 2.3.2. Realization of Reverse Bias at the Base–Collector 

Similarly, the intrinsic carrier concentration distribution of P_2_-type region (P_2_-Si) under the applied temperature gradient can be expressed [25]:

(32) $$p_{i2}\left(x_{2}\right)=5.23\times 10^{15}\times {T_{2}\left(x_{2}\right)}^{\frac{3}{2}}\times \exp\left(\frac{6395.39}{T_{2}\left(x_{2}\right)}\right)$$

In order ensure the normal operation of the thermoelectric transistor, the base–collector junction should be the reverse bias. Under the influence of the temperature distribution *T*_2_(*x*_2_), the built-in voltage at the base–collector junction can be expressed as:

(33) $$V_{bi2}\left(x_{2}\right)=\frac{k_{B}T\left(x_{2}\right)}{q}\left[\ln\frac{P_{a2}}{p_{i2}\left(x_{2}\right)}+\ln\frac{N_{d}}{n_{i}\left(x_{2}\right)}\right]$$

To ensure proper operation, *V_bi_*_2_(*x*_2_) must be positive:

(34) $$V_{bi2}\left(x_{2}\right)>0$$

Similar to the emitter–base junction, the potential of the base should be higher than that of the collector:

(35) $$\varphi_{n}\left(x_{2}\right)>\varphi_{p2}\left(x_{2}\right)$$

(36) $$N_{d}>p_{2}\left(x_{2}\right)$$

Furthermore, the Seebeck coefficient and the Seebeck voltage for P_2_-Si are expressed as follows:

(37) $$V_{s2}\left(x_{2}\right)=∆T\left(x_{2}\right)\times S_{2}$$

(38) $$S_{2}=\frac{k_{B}}{e}\left[\left(r+\frac{5}{2}\right)+\ln\frac{2{\left(2\pi m_{p2}^{*}k_{B}T_{2}\left(x_{2}\right)\right)}^{3/2}}{h^{3}p_{2}\left(x_{2}\right)}\right]$$

where *p*_2_(*x*_2_) is the hole concentration distribution in the collector region after applying the temperature gradient. 

By solving these equations, the operation conditions of the base–collector junction can be determined.

### 2.4. The Output Performance of Thermoelectric Transistors

**Figure 2:**
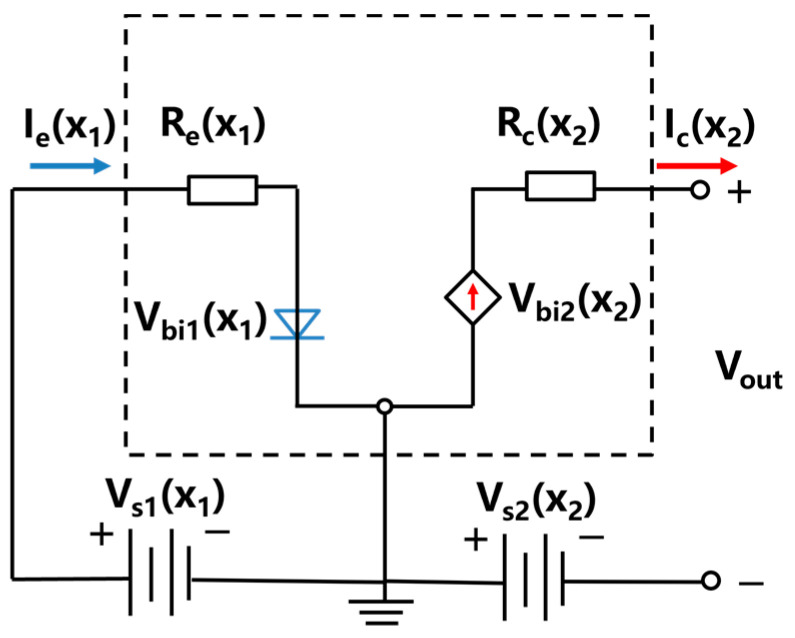
Schematic diagram of equivalent circuit in thermoelectric transistor.

For bipolar thermoelectric transistors, the base–collector circuit can be considered as the output circuit. In this work, the early voltage effects of the emitter, base, and collector, as well as the contact resistance between the transistor and the electrodes, can be neglected. The corresponding equivalent DC circuit diagram is shown in Figure 2. The left side of the circuit diagram represents the equivalent circuit of the emitter–base junction. *V_s_*_1_(*x*_1_) denotes the Seebeck voltage generated in the P_1_ region, serving as the external voltage at the emitter. *R_e_*(*x*_1_) represents the resistance of the P_1_ region of the emitter. *V_bi_*_1_(*x*_1_) is assumed to be the forward voltage. On the right side of the circuit, the equivalent circuit of the base–collector junction is shown. *V_s_*_2_(*x*_2_) signifies the Seebeck voltage generated in the P_2_ region, acting as the external voltage at the collector. *R_c_*(*x*_2_) corresponds to the resistance of the P_2_ region of the collector. *V_bi_*_2_(*x*_2_) is the voltage influenced by the emitter current *I_e_*(*x*_1_). Furthermore, considering that the thickness of the base can be neglected compared to the emitter and collector and that it is fully depleted, the collector current *I_c_*(*x*_2_) can be assumed to be equal to the emitter current *I_e_*(*x*_1_).

According to the Kirchhoff’s law, the relationships among these electrical parameters can be expressed:

(39) $$V_{e}\left(x_{1}\right)=V_{s1}\left(x_{1}\right)-V_{bi1}\left(x_{1}\right)=I_{e}\left(x_{1}\right)R_{e}\left(x_{1}\right)$$

(40) $$V_{c}\left(x_{2}\right)=V_{bi2}\left(x_{2}\right)-V_{s2}\left(x_{2}\right)=I_{c}\left(x_{2}\right)R_{c}\left(x_{2}\right)$$

(41) $$I_{e}\left(x_{1}\right)=I_{c}\left(x_{2}\right)$$

(42) $$R_{e}\left(x_{1}\right)=\frac{1}{p_{a1}e\mu_{p1}}\times \frac{l_{x1}}{w_{y}h_{z}}$$

(43) $$R_{c}\left(x_{2}\right)=\frac{1}{p_{a2}e\mu_{p2}}\times \frac{l_{x2}}{w_{y}h_{z}}$$

where *V_e_*(*x*_1_) is the emitter voltage, *V_c_*(*x*_2_) is the collector voltage, and *μ_p_*_1_ and *μ_p_*_2_ are the hole mobility of the P_1_-type material (P_1_-Bi_2_Te_3_) and P_2_-type material (P_2_-Si), respectively. *lx*_1_ and *lx*_2_ represent the lengths of the P_1_-type and P_2_-type materials along the *x* direction, while *w_y_* and *h_z_* denote the lengths of the device in *y* and *z* directions, respectively, with values set to 1 μm. *S_p_*_2_ is the Seebeck coefficient of the collector and m*_p__2_ is the effective mass of the collector. 

The output power of the transistor can be expressed as the product of the open-circuit voltage and the short-circuit current:

(44) $$P_{out}=I_{e}\left(x_{1}\right)\times V_{out}=I_{e}\left(x_{1}\right)\times V_{c}\left(x_{2}\right)=I_{e}\left(x_{1}\right)\times [V_{bi2}\left(x_{2}\right)-V_{s2}\left(x_{2}\right)]$$

### 2.5. The Material Parameters of Thermoelectric Transistor

In this work, P_1_-Bi_2_Te_3_, N-Si, and P_2_-Si are selected as the emitter material, base material, and collector material, respectively. The material parameters of the thermoelectric transistor studied are listed in Table 1. 

## 3. Results and Discussion

### 3.1. Temperature Distribution in Thermoelectric Transistor

#### 3.1.1. Temperature Distribution in p-Type Bi_2_Te_3_

**Figure 3:**
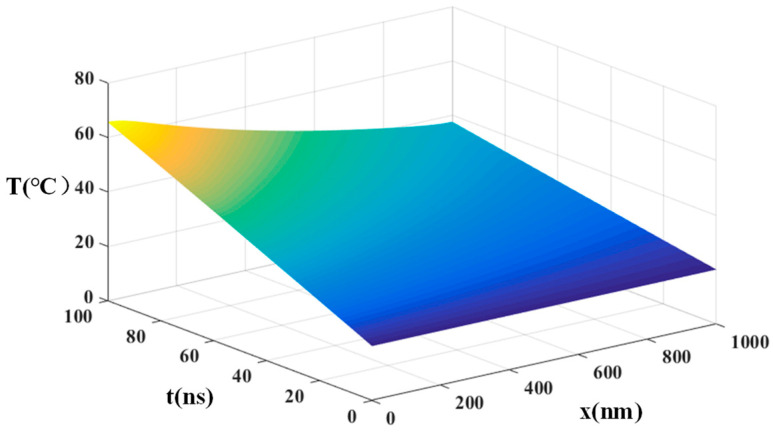
Temperature distribution of Bi_2_Te_3_ after laser irradiation with time *t* and *x*.

It is assumed that P_1_-Bi_2_Te_3_ is in the temperature of 20 °C. Under the condition of illuminating with an incident optical power of =3 × 10^7^(W·cm^−2^) for a duration of 100 ns, the temperature distribution within Bi_2_Te_3_ is obtained as shown in Figure 3.

**Figure 4:**
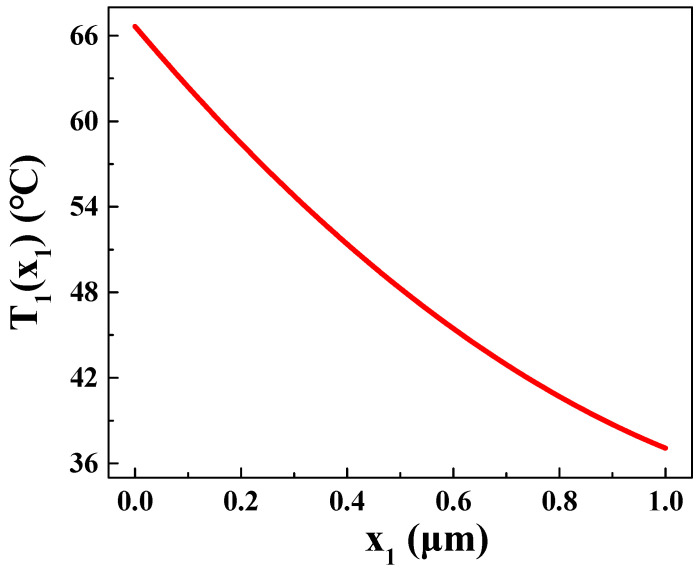
Temperature distribution in P_1_ region at *t* = 100 ns.

As depicted in Figure 3, it is apparent that, with an increase in laser irradiation time, the sample initiates heating from the irradiated surface at *x* = 0 μm. Subsequently, the heat gradually propagates throughout the entire device, and the lowest temperature is observed at *x* = 1 μm within P_1_ region. Figure 4 illustrates the temperature distribution in P_1_ region at *T* = 100 ns. Notably, after 100 ns of illumination, the temperature distribution in P_1_ region (Bi_2_Te_3_) exhibits a pattern of decreasing temperature as the distance from the laser increases. Specifically, at *x* = 0 μm, the temperature at 100 ns is *T*_b1_ = 66.7 °C, whereas, at *x* = 1 μm, the temperature reaches its minimum value of *T*_b2_ = 37 °C. By employing a quadratic function, the temperature variation *T*_1_(*x*_1_) in P_1_ region as a function of *x* can be obtained:

(45) $$T_{1}\left(x_{1}\right)=14.35x_{1}^{2}-43.95x_{1}+66.7$$

#### 3.1.2. Temperature Distribution in p-Si

**Figure 5:**
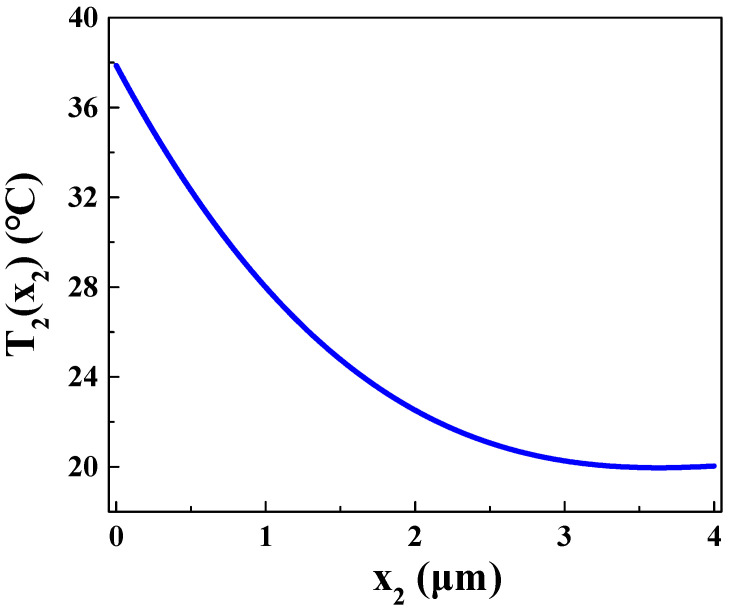
Temperature distribution in P_2_ region at *t* = 100 ns.

By solving the equations in MATLAB, the temperature distribution in P_2_ region (P_2_-Si) along the *x* direction can be obtained. After 100 ns, the temperature distribution is shown in Figure 5.

Based on Figure 5, it can be observed that the temperature distribution in P_2_-type region gradually decreases with increasing *x*. It is important to note that n-type region in this work is extremely thin. Therefore, the length of the n-type region along the *x* direction is assumed to be 0, implying that *x*_1_ = 1 μm coincides with *x*_2_ = 0 μm. At *x*_2_ = 0 μm, P_2_-Si exhibits the highest temperature, *T_s_*_1_ = 37 °C, while, at *x*_2_ = 4 μm, p-Si reaches its lowest temperature, *T_s_*_2_ = 20 °C. By fitting a curve to the data in the figure, a fitting function *T*_2_(*x*_2_) for the temperature distribution in P_2_-Si can be obtained:

(46) $$T_{2}\left(x_{2}\right)=-0.1982x_{2}^{3}+{2.798x}_{2}^{2}-12.48x_{2}+37$$

### 3.2. Hole Concentration Distribution within Thermoelectric Transistor

The thermoelectric transistor consists of P_1_-type region, N-type region, and P_2_-type region. As mentioned in the previous section, the temperature distribution along the *x* direction of the transistor is represented by Equations (45) and (46).

Considering that N-type region is assumed to be completely depleted, it contains no free mobile carriers. However, for both P_1_-type region and P_2_-type region, under the influence of temperature distribution, the holes migrate from the hot end to the cold end, leading to their accumulation at the cold end. By utilizing the calculation formula from the previous section, the concentration distribution of holes in P_1_-type region can be obtained:

(47) $$p_{1}\left(x_{1}\right)=\frac{P_{a1}\times \left(-6.429\times 10^{-13}\right)}{{\left(x_{1}-1.532\times 10^{-4}\right)}^{3}}$$

**Figure 6:**
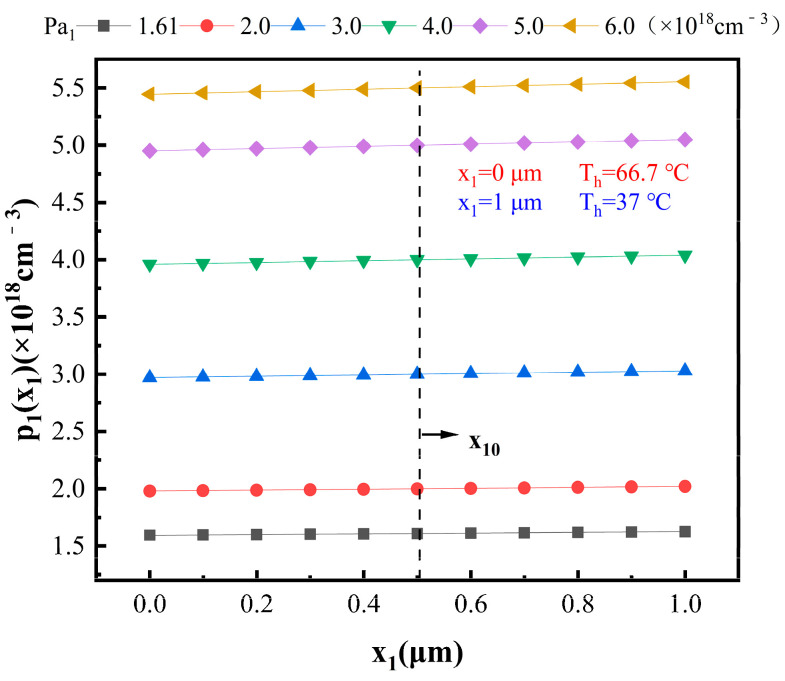
Relationship between *p*_1_(*x*_1_) and *x*_1_ at different hole concentrations.

From Figure 6, it is evident that *p*_1_(*x*_1_) increases with increasing *x*_1_. This indicates that the hole concentration in P_1_-type region gradually increases from the hot end to the cold end. Additionally, as *P_a_*_1_ increases, *p*_1_(*x*_1_) also increases. Of note, *p*_1_(*x*_1_) = *P_a_*_1_ is near the midpoint of the P_1_-type region (*x*_10_ = 0.501 μm), as shown in the dashed line in Figure 6. This is because the hole carriers in the P_1_-type region (Bi_2_Te_3_) redistribute only along the *x* direction, and the temperature difference is fixed. Therefore, the position *x*_10_ remains constant. As a result, it can be inferred that, for *x*_1_ < *x*_10_, *p*_1_(*x*_1_) < *P_a_*_1_, and, for *x*_10_ ≤ *x*_1_ ≤ 1 μm, *p*_1_(*x*_1_) ≥ *P_a_*_1_, with equality only at *x*_1_ = *x*_10_.

Similarly, hole concentration distribution of P_2_-Si can be obtained:

(48) $$p_{2}\left(x_{2}\right)=\frac{P_{a2}\times \left(5.773\times 10^{4}\right)}{{\left(1.858\times 10^{8}\times x_{2}^{2}-1.749\times 10^{5}\times x_{2}+39\right)}^{3}}$$

**Figure 7:**
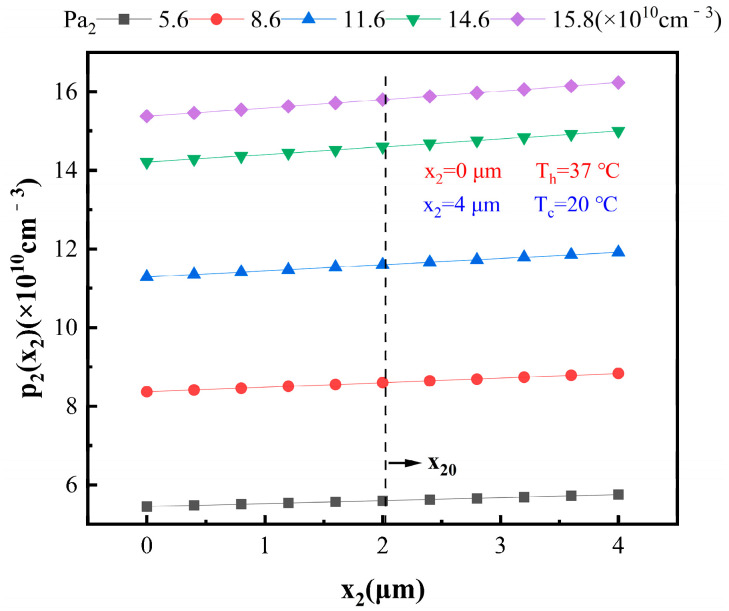
Relationship between *p*_2_(*x*_2_) and *x*_2_ at different hole concentrations.

Figure 7 illustrates the variation in *p*_2_(*x*_2_) with respect to *x*_2_ for different *P_a_*_2_. It can be observed that *p*_2_(*x*_2_) also increases with the increase in *x*_2_. Additionally, as *P_a_*_2_ increases, *p*_2_(*x*_2_) increases. Similarly, at *x*_20_ = 2.009 μm, *p*_2_(*x*_2_) equals *P_a_*_2_, as indicated by the dashed line in Figure 7. In other words, for 0 ≤*x*_2_ < *x*_20_ = 2.009 μm, *P_a_*_2_ > *p*_2_(*x*_2_), and, for *x*_20_ ≤ *x*_2_ ≤ 4 μm, *p*_2_(*x*_2_) ≥ *P_a_*_2_, with equality only at *x*_2_ = *x*_20_.

Thus, it can be inferred that the Seebeck effect causes the redistribution of hole concentrations in both P_1_-type and P_2_-type regions, with holes diffusing from the hot end to the cold end. Consequently, the built-in voltage at both the P_1_–N junction and the N–P_2_ junction decreases from the cold end to the hot end. This unique characteristic enables such PNP heterojunctions to function as thermoelectric transistors. The emitter corresponds to the region with *x*_10_ ≤ *x*_1_ ≤ 1 μm in the P_1_-type region, the base corresponds to the entire N-type region, and the collector corresponds to the region with 0 ≤ *x*_2_ < *x*_20_ = 2.009 μm in the P_2_-type region.

### 3.3. Operarion Conditions in Thermoelectric Transistor

#### 3.3.1. Forward Bias of Emitter–Base Junction

When the entire thermoelectric transistor is at 20 °C, based on Equations (20)–(22), the required values for *P_a_*_1_, *N_d_*, and *P_a_*_2_ can be determined:

(49) $$P_{a1}>p_{i1}=1.609\times 10^{18}\left({cm}^{-3},T=20\;{}^{\circ}C\right)$$

(50) $$N_{d}>n_{i}=3.179\times 10^{10}\left({cm}^{-3},T=20\;{}^{\circ}C\right)$$

(51) $$P_{a2}>p_{i2}=9.268\times 10^{9}\left({cm}^{-3},T=20\;{}^{\circ}C\right)$$

For simplicity, their values are assumed to be 1.61 × 10^18^ cm^−3^, 3.2 × 10^10^ cm^−3^, and 1.0 × 10^10^ cm^−3^, respectively.

The temperature distribution *T*_1_(*x*_1_) and the temperature difference Δ*T*_1_(*x*_1_) for the emitter–base junction can be written:

(52) $$T_{1}\left(x_{1}\right)=14.35x_{1}^{2}-43.95x_{1}+66.7$$

(53) $${∆T}_{1}\left(x_{1}\right)=-14.35x_{1}^{2}-43.95x_{1}$$

(54) $$x_{1}\in \left[0.501,1\right]\;\left(\mu m\right)$$

**Figure 8:**
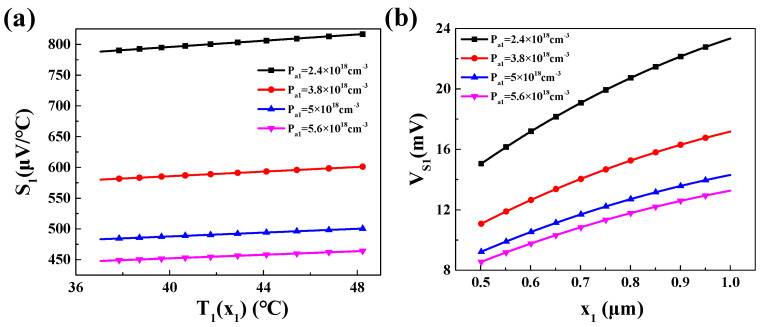
In the P_1_ region, under different doping concentrations *P_a_*_1_: (**a**) the relationship between Seebeck coefficient *S*_1_ and temperature; (**b**) variation in Seebeck voltage *V_s_*_1_ at different *x*_1_ positions.

Under this temperature distribution, the calculated values for the Seebeck coefficient *S*_1_ and the Seebeck voltage *V_s_*_1_ in P_1_ region are shown in Figure 8.

As shown in Figure 8, the Seebeck coefficient *S*_1_ of P_1_ region increases with the temperature *T*_1_(*x*_1_). Moreover, for different doping concentrations *P_a_*_1_, the smaller doping concentration leads to a larger Seebeck coefficient. As for the Seebeck voltage *V_s_*_1_, it exhibits a trend where it increases as the distance from the hot end (*x*_1_ = 0) increases due to the larger temperature difference. 

**Figure 9:**
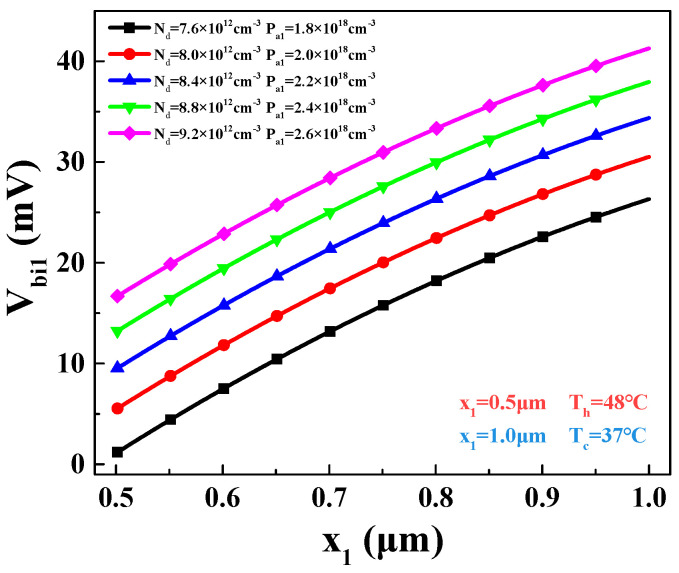
Different values of built-in voltage in P_1_-N region on different *x*_1_.

Additionally, the built-in voltage *V_bi_*_1_ within P_1_-N region can be obtained, as shown in Figure 9.

Under different values of *P_a_*_1_ and *N_d_*, the built-in voltage increases with the increase in *x*_1_. This means that, as the temperature decreases, the built-in voltage increases. The increase is primarily attributed to the decrease in intrinsic carrier concentration with decreasing temperature.

**Figure 10:**
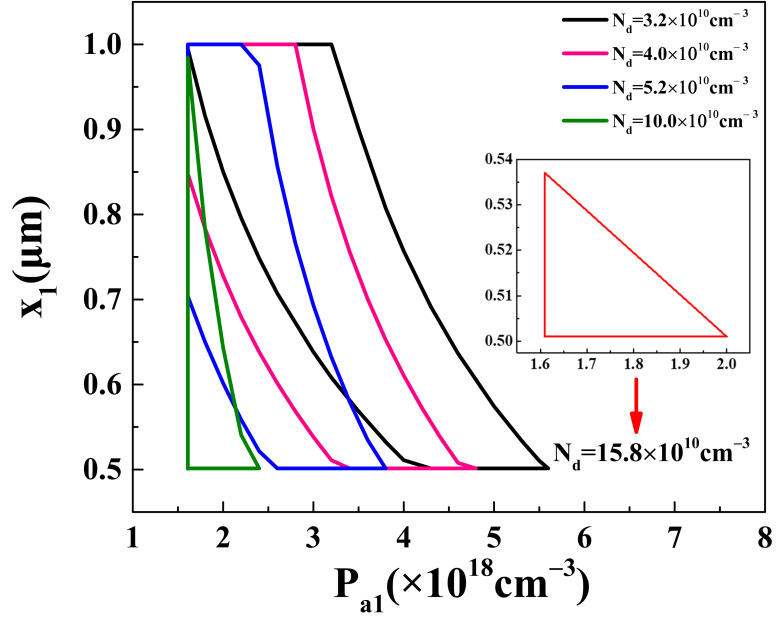
Suitable range and corresponding *x*_1_ value of *P_a_*_1_ that satisfies forward bias and forward conduction of emitter–base.

Based on Section 2.3.1, it can be concluded that achieving the forward bias and forward conduction of the emitter–base junction depends on three conditions: *V_bi_*_1_(*x*_1_) > 0, *V_e_*(*x*_1_) = *V_s_*_1_(*x*_1_)–*V_bi_*_1_(*x*_1_) > 0, and *p*_1_(*x*_1_) > *N_d_*. These conditions are all influenced by *P_a_*_1_, *x*_1_, and *N_d_*. The appropriate concentration ranges for *P_a_*_1_ and *N_d_* are shown in Figure 10.

It can be observed that the suitable range for *N_d_* is 3.2 × 10^10^ cm^−3^~15.8 × 10^10^ cm^−3^. As the range of *N_d_* increases, the range of *P_a_*_1_ decreases. When *N_d_* is 3.2 × 10^10^ cm^−3^, the range of *P_a_*_1_ is 1.61 × 10^18^ cm^−3^~5.6 × 10^18^ cm^−3^, as indicated by the black line. When *N_d_* increases to 15.8 × 10^10^ cm^−3^, the range of *P_a_*_1_ is only 1.61 × 10^18^ cm^−3^~2.0 × 10^18^ cm^−3^, as shown by the red line. This is because the built-in voltage *V_bi_*_1_(*x*_1_) is positively correlated with *N_d_* and *P_a_*_1_. As *N_d_* and *P_a_*_1_ increase, *V_bi_*_1_(*x*_1_) also increases, resulting in a smaller range of *P_a_*_1_ for achieving *V_s_*_1_(*x*_1_)–*V_bi_*_1_(*x*_1_) > 0.

Additionally, it can be found that *x*_1_ also changes with *N_d_* and *P_a_*_1_. As *N_d_* and *P_a_*_1_ increase, the range of *x*_1_ gradually approaches 0.501 μm. This is because, at *x*_1_ = 1 μm, both *V_bi_*_1_(*x*_1_) and *V_s_*_1_(*x*_1_) reach their maximum values. However, in comparison to *V_s_*_1_(*x*_1_), *V_bi_*_1_(*x*_1_) increases at a faster rate as *x*_1_ increases. Therefore, it is relatively easier to achieve the operation conditions of the emitter–base junction at *x*_1_ = 0.501 μm. Thus, when *N_d_* = 3.2 × 10^10^ cm^−3^, the maximum range of *x_1_* is 0.608 μm to 1 μm. While *N_d_* = 15.8 × 10^10^ cm^−3^, the maximum range of *x*_1_ is 0.501 μm to 0.537 μm. Therefore, the forward bias and forward conduction of the emitter–base junction can be achieved by adjusting the concentrations of *P_a_*_1_ and *N_d_*. Such a deigned doping concentration for P_1_-Bi_2_Te_3_ can be obtained directly by doping process, e.g., phosphorus as acceptor.

#### 3.3.2. Reverse Bias of Base–Collector Junction

When a temperature gradient is applied in the base–collector junction, the temperature distribution equation *T*_2_(*x*_2_) and temperature difference ∆*T*_2_(*x*_2_) can be expressed as follows:

(55) $$T_{2}\left(x_{2}\right)=-0.1982x_{2}^{3}+{2.798x}_{2}^{2}-12.48x_{2}+37$$

(56) $${∆T}_{2}\left(x_{2}\right)=0.1982x_{2}^{3}-{2.798x}_{2}^{2}+12.48x_{2}-17$$

(57) $$x_{2}\in \left[0,0.2\right]\;\left(\mu m\right)$$

It should be noted that ∆*T*(*x*_1_) represents the temperature difference between the emitter and base, with a positive value. However, ∆*T*_2_(*x*_2_) represents the temperature difference between the collector and base, with a negative value. Therefore, *V_s_*_1_(*x*_1_) has a positive value, while *V_s_*_2_(*x*_2_) has a negative value. 

**Figure 11:**
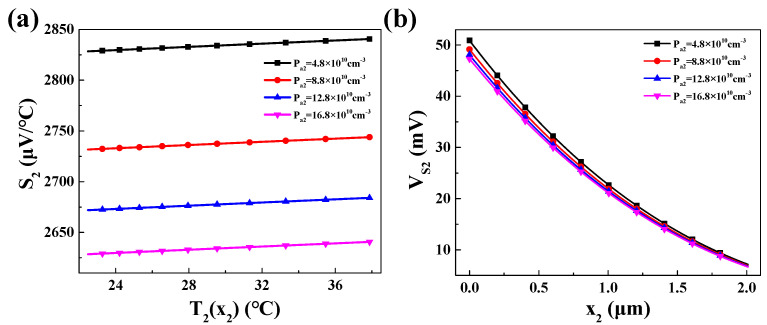
Under different doping concentrations *P_a_*_2_: (**a**) the relationship between Seebeck coefficient *S*_2_ and temperature; (**b**) variation in Seebeck voltage *V_s_*_2_ at different *x*_2_ positions.

For P_2_ region, the values of the Seebeck coefficient *S*_2_ and the Seebeck voltage *V_s_*_2_ are shown in Figure 11.

It can be observed that the Seebeck coefficient *S*_2_ of P_2_ region increases with temperature *T*_2_(*x*_2_). For different doping concentrations *P_a_*_2_, a smaller doping concentration leads to a larger Seebeck coefficient. Regarding the Seebeck voltage *V_s_*_2_, it exhibits a trend as the position approaches *x*_2_ = 0 (closer to the hot end) due to the increasing temperature difference. 

**Figure 12:**
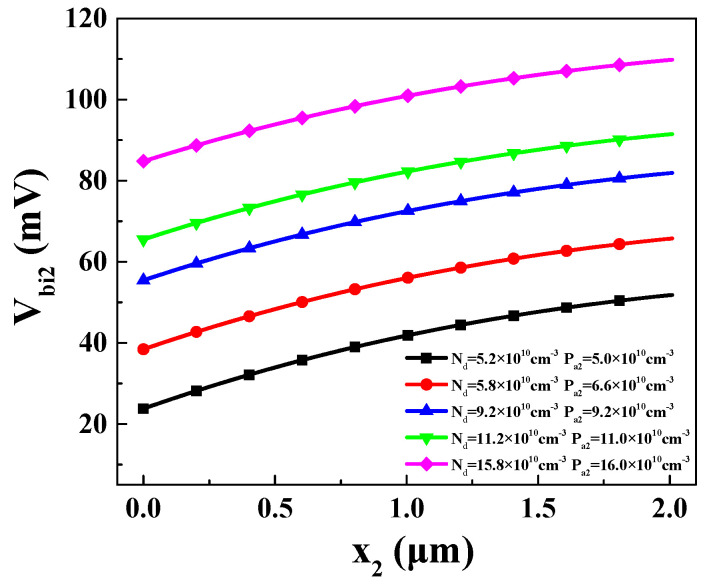
Different values of built-in voltage in P_2_-N region on different *x*_2_.

The built-in voltage *V_bi_*_2_ in the N-P_2_ region is depicted in Figure 12. It can be found that the built-in voltage *V_bi_*_2_ increases with increasing *x*_2_ at different values of *P_a_*_2_ and *N_d_.* In other words, as the temperature decreases, the built-in voltage increases. This increase primarily stems from the decrease in intrinsic charge carriers with decreasing temperature. 

**Figure 13:**
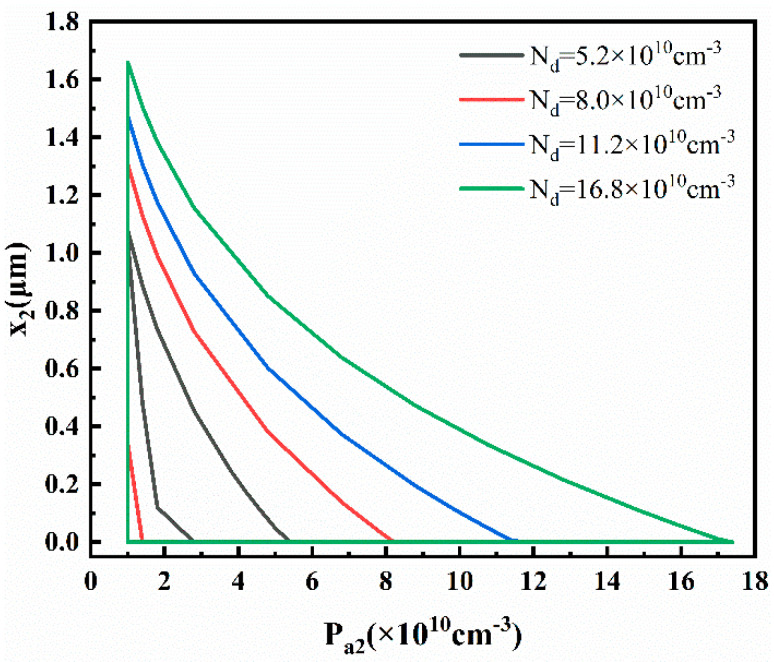
Suitable range and corresponding *x*_2_ value of *P_a_*_2_ that satisfies forward bias and forward conduction of emitter–base.

Based on Section 2.3.2, it can be concluded that achieving the forward bias and forward conduction of the emitter–base junction depends on three conditions: *V_bi_*_2_(*x*_2_) > 0 and *p*_2_(*x*_2_) < *N_d_*. These conditions are all influenced by *P_a_*_2_, *x*_2_, and *N_d_*. Therefore, the reverse bias in the base–collector junction can be achieved by adjusting the concentrations of *P_a2_* and *N_d_*. The appropriate concentration ranges for *P_a_*_2_ and *N_d_* are shown in Figure 13.

Unlike the previous section, the range of *N_d_* is from 5.2 × 10^10^ cm^−3^ to 15.8 × 10^10^ cm^−3^. It can be observed that, as *N_d_* increases, the corresponding range of *P_a2_* also gradually increases. This is because higher values of *N_d_* and *P_a_*_2_ both lead to larger values of *V_bi_*_2_(*x*_2_), making it easier to satisfy the reverse biasing condition *V_bi_*_2_(*x*_2_) > 0.

To summarize, when *N_d_* is within the range of 5.2 × 10^10^ cm^−3^ to 15.8 × 10^10^ cm^−3^, the thermoelectric transistor can be activated in the forward-active mode. The corresponding ranges of *P_a_*_1_, *P_a_*_2_, *x*_1_, and *x*_2_ are determined by the different values of *N_d_*. By adjusting the concentrations of *P_a_*_1_, *N_d_*, and *P_a_*_2_, the current can be generated in the thermoelectric transistor directly driven by the Seebeck effect.

### 3.4. Output Performance of Thermoelectric Transistor

It is evident that the output power of the transistor is primarily influenced by *P_a_*_1_, *N_d_*, *P_a_*_2_, *x*_1_, and *x*_2_ based on Equation (44). Therefore, the impact of these parameters on the output power of the thermoelectric transistor is obtained in the following.

#### 3.4.1. Impact of N_d_ on Output Power of Thermoelectric Transistor

**Figure 14:**
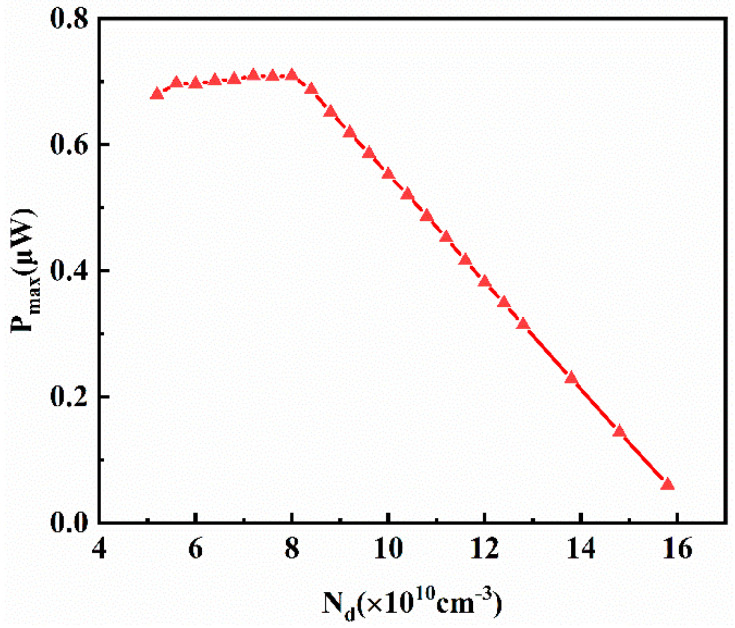
Variation in the maximum output power *P_max_* of the transistor with the value of *N_d_*.

Since the value of *N_d_* directly affects the other four physical quantities, it is essential to initially examine the influence of *N_d_* on the output power. Figure 14 illustrates the relationship between *N_d_* and the maximum output power of the thermoelectric transistor.

It is evident that, as *N_d_* gradually increases, *P_outmax_* exhibits a trend of initially increasing and then decreasing. At *N_d_* = 8.0 × 10^10^ cm^−3^, *P_outmax_* reaches its maximum value of 0.7093 μW. It is important to note that different values of *N_d_* correspond to various combinations of *P_a_*_1_, *P_a_*_2_, *x*_1_, and *x*_2_. Considering the multitude of possible parameter combinations, it is challenging to directly explain how *N_d_* influences the output power. Therefore, a comprehensive enumeration of the parameters is necessary to identify the optimal solution, which, in this case, is *N_d_* = 8.0 × 10^10^ cm^−3^ and *P_outmax_* = 0.7093 μW.

#### 3.4.2. Impact of P_a1_ and x_1_ on Output Power of Thermoelectric Transistor

**Figure 15:**
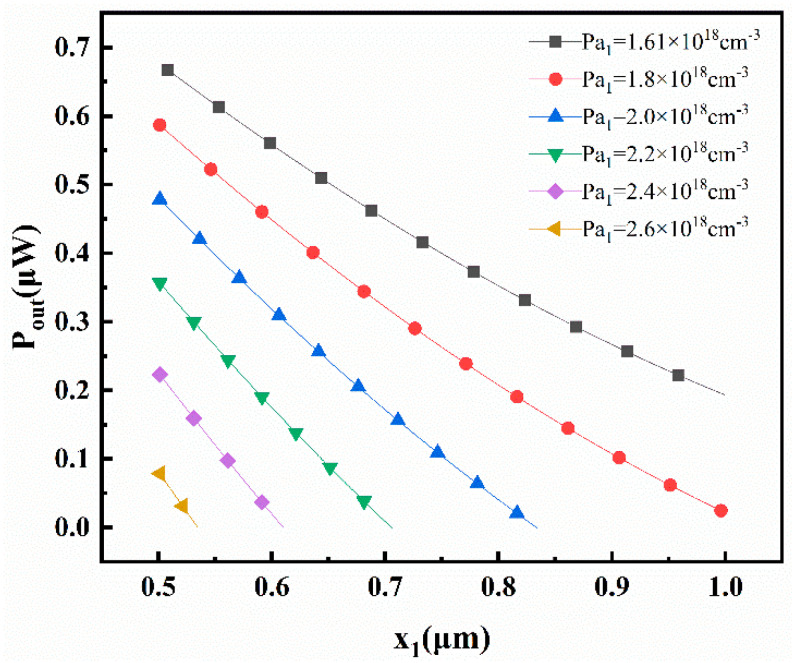
Variation in the maximum output power of transistor *P_out_* with the values of *P_a_*_1_ and *x*_1_.

For *N_d_* = 8.0 × 10^10^ cm^−3^, the range of *P_a1_* is from 1.61 × 10^18^ cm^−3^ to 2.6 × 10^18^ cm^−3^ and the range of *P_a_*_2_ is from 1.0 × 10^10^ cm^−3^ to 8.2 × 10^10^ cm^−3^. Moreover, different values of *P_a_*_1_ and *P_a_*_2_ correspond to specific ranges of *x*_1_ and *x*_2_. Considering a fixed *P_a_*_2_ value of 7.8 × 10^10^ cm^−3^ and *x*_2_ value of 0.4 μm, the relationship between output power (*P_out_*) and the variations in *P_a_*_1_ and *x*_1_ is illustrated in Figure 15.

It can be observed that the output power (*P_out_*) increases as the concentration of *P_a_*_1_ and *x_1_* decrease. These results can be derived from Equation (58):

(58) $$P_{out}=I_{e}\left(x_{1}\right)\times [V_{bi2}\left(x_{2}\right)-V_{s2}\left(x_{2}\right)]$$

When *N_d_*, *P_a_*_2_, and *x*_2_ remain constant, the value of *P_out_* is primarily influenced by *I_e_*(*x*_1_):

(59) $$I_{e}\left(x_{1}\right)=\frac{V_{s1}\left(x_{1}\right)-V_{bi1}\left(x_{1}\right)}{R_{e}\left(x_{1}\right)}$$

At *x*_1_ = 1 μm, both *V_bi_*_1_(*x*_1_) and *V_s_*_1_(*x*_1_) reach their maximum values. However, compared to *V_s_*_1_(*x*_1_), *V_bi_*_1_(*x*_1_) increases more rapidly as *x*_1_ increases. Therefore, at *x*_1_ = 0.501 μm, *V_s_*_1_(*x*_1_)–*V_bi_*_1_(*x*_1_) reaches its maximum value. Thus, as *x*_1_ decreases within the range of *x*_1_ ∈ (0.501 μm, 1 μm), *P_out_* increases. On the other hand, when other conditions remain unchanged, a larger *P_a_*_1_ leads to a higher built-in voltage in the P_1_ region (*V_s_*_1_(*x*_1_)–*V_bi_*_1_(*x*_1_)). As a result, with an increase in *P_a_*_1_, *P_out_* decreases.

#### 3.4.3. Impact of P_a2_ and x_2_ on Output Power of Thermoelectric Transistor

**Figure 16:**
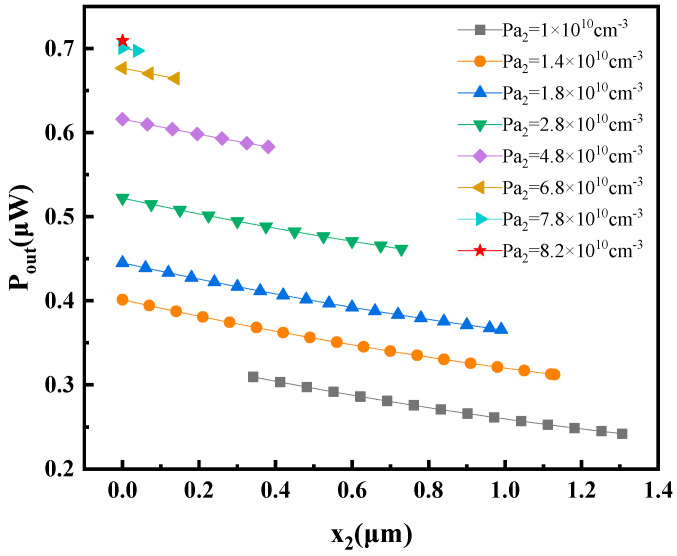
Variation in the maximum output power of transistor *P_out_* with the values of *P_a_*_2_ and *x*_2_.

The impact of *P_a_*_2_ and *x*_2_ on the output power of the thermoelectric transistor is examined in the following when *P_a_*_1_ = 1.61 × 10^18^ cm^−3^ and *x*_1_ = 0.508 μm. As shown in Figure 16, it can be observed that *P_out_* increases as *P_a_*_2_ increases and *x*_2_ decreases. Again, referring to the expression for *P_out_*:

(60) $$P_{out}=I_{e}\left(x_{1}\right)\times [V_{bi2}\left(x_{2}\right)-V_{s2}\left(x_{2}\right)]$$

When *N_d_*, *P_a_*_1_, and *x*_1_ are constant, the value of *P_out_* is mainly influenced by *V_bi_*_2_(*x*_2_)–*V_s_*_2_(*x*_2_). An increase in *P_a_*_2_ leads to a higher value of *V_bi_*_2_(*x*_2_), resulting in a larger difference between *V_bi_*_2_(*x*_2_) and *V_s_*_2_(*x*_2_). Consequently, as *P_a_*_2_ increases, *P_out_* also increases. On the other hand, when *x*_2_ increases, ∆*T*_2_(*x*_2_) decreases, resulting in a lower *V_s_*_2_(*x*_2_) and higher *V_bi_*_2_(*x*_2_). In this case, *V_s_*_2_(*x*_2_) dominates the relationship and the value of *V_bi_*_2_(*x*_2_)–*V_s_*_2_(*x*_2_) decreases, leading to a decrease in *P_out_*.

#### 3.4.4. Transistor Structure: Optimization of Output Power Effects

As deduced from the above analysis, when *N_d_* = 8.0 × 10^10^ cm^−3^, *P_a_*_1_ = 1.61 × 10^18^ cm^−3^, *P_a_*_2_ = 8.2 × 10^10^ cm^−3^, *x*_1_ = 0.508 μm, and *x*_2_ = 0 μm, the maximum output power of this designed thermoelectric transistor can reach 0.7093 μW.

If only Bi_2_Te_3_ is used without the thermoelectric transistor structure, the output power, can be calculated as 0.1442 μW. It can be observed that the designed thermoelectric transistor structure in this work results in an increase in the output power compared to a single thermoelectric material, with an improvement of 391.9%. 

## 4. Conclusions

In this work, the temperature distribution and hole concentration distribution within the thermoelectric transistor is obtained through theoretical calculations. By considering the variation in the built-in voltage with temperature, the formation principle of the thermoelectric transistor is demonstrated. Subsequently, based on the operation conditions of the thermoelectric transistor, suitable doping concentrations of the emitter, base, and collector can be determined. Furthermore, the maximum output power of the thermoelectric transistor is obtained. The main conclusions drawn from this work are as follows:

(i) The thermoelectric transistor, composed of P_1_-Bi_2_Te_3_, N-Si, and P_2_-Si, is directly irradiated by the laser. Under laser illumination and heat conduction, the temperature decreases from 66.7 °C to 20 °C, creating a temperature difference of 46.7 °C at the two ends of the thermoelectric transistor. As a result of the temperature difference, holes inside P_1_ and P_2_ regions migrate from the hot end to the far end, leading to increased hole concentration at the cold end.
(ii) The operation conditions of the thermoelectric transistor under laser irradiation are investigated. Based on the corresponding conditions, suitable doping concentrations of the emitter, base, and collector can be determined. By adjusting these concentrations, the current can be produced in the thermoelectric transistor only driven by the Seebeck effect.
(iii) The influence of these parameters on the output power of the thermoelectric transistor is also investigated. The maximum output power of the thermoelectric transistor is 0.7093 μW under a temperature difference of 46.7 °C, which is nearly quadrupling the performance compared to the single thermoelectric material structure.
(iv) Importantly, the operation conditions of the thermoelectric transistor established in this work are applicable to other material systems. By adjusting the doping concentration within each region, current can be generated, ensuring that the forward-active mode is achieved. Therefore, this novel thermoelectric generator concept can significantly contribute to the advancement of the thermoelectric field. Moreover, the combination with transistor technology can expand the range of applications for thermoelectric generators.

## Figures and Tables

**Table 1:** Material parameters of thermoelectric transistor.

Parameter	Emitter	Base	Collector	Ref.
E_g_ (eV)	0.18	1.12	1.12	[25,27]
m*/m_e_	1.4671	0.43	0.2601	[28,29,30]
ε_r_	90	11.7	11.7	[6,25]
μ_p_ (cm^2^/V s)	510	-	480	[21,25]

## Data Availability

The data presented in this study are available upon request from the corresponding author.
